# Deleted in Malignant Brain Tumors 1 (DMBT1) is present in hyaline membranes and modulates surface tension of surfactant

**DOI:** 10.1186/1465-9921-8-69

**Published:** 2007-10-01

**Authors:** Hanna Müller, Caroline End, Marcus Renner, Burkhard M Helmke, Nikolaus Gassler, Christel Weiss, Dominik Hartl, Matthias Griese, Mathias Hafner, Annemarie Poustka, Jan Mollenhauer, Johannes Poeschl

**Affiliations:** 1 Division of Neonatology, Department of Pediatrics, University of Heidelberg, Im Neuenheimer Feld 153, 69120 Heidelberg, Germany; 2 Division of Molecular Genome Analysis, German Cancer Research Center, Im Neuenheimer Feld 280, 69120 Heidelberg, Germany; 3 Institute of Molecular Biology and Cell Culture Technology, University of Applied Sciences Mannheim, 68163 Mannheim, Germany; 4 Institute of Pathology, University of Heidelberg, Im Neuenheimer Feld 220/221, 69120 Heidelberg, Germany; 5 Institute of Pathology, University Hospital, RWTH Aachen, Pauwelsstrasse 30, 52074 Aachen, Germany; 6 Institute of Medical Statistics and Biomathematics, University Hospital Mannheim, Theodor-Kutzer-Ufer 1, 68135 Mannheim, Germany; 7 Children's Hospital, University of Munich, Lindwurmstrasse 2a, 80337 Munich, Germany

## Abstract

**Background:**

Deleted in Malignant Brain Tumors 1 (DMBT1) is a secreted scavenger receptor cysteine-rich protein that binds various bacteria and is thought to participate in innate pulmonary host defense. We hypothesized that pulmonary DMBT1 could contribute to respiratory distress syndrome in neonates by modulating surfactant function.

**Methods:**

DMBT1 expression was studied by immunohistochemistry and mRNA *in situ *hybridization in post-mortem lungs of preterm and full-term neonates with pulmonary hyaline membranes. The effect of human recombinant DMBT1 on the function of bovine and porcine surfactant was measured by a capillary surfactometer. DMBT1-levels in tracheal aspirates of ventilated preterm and term infants were determined by ELISA.

**Results:**

Pulmonary DMBT1 was localized in hyaline membranes during respiratory distress syndrome. *In vitro *addition of human recombinant DMBT1 to the surfactants increased surface tension in a dose-dependent manner. The DMBT1-mediated effect was reverted by the addition of calcium depending on the surfactant preparation.

**Conclusion:**

Our data showed pulmonary DMBT1 expression in hyaline membranes during respiratory distress syndrome and demonstrated that DMBT1 increases lung surface tension *in vitro*. This raises the possibility that DMBT1 could antagonize surfactant supplementation in respiratory distress syndrome and could represent a candidate target molecule for therapeutic intervention in neonatal lung disease.

## Background

Respiratory distress syndrome (RDS) in neonates due to surfactant deficiency has been shown to be associated with a strong immune response such as activation of complement [1] and neutrophils [2]. Further, ventilation of preterm infants with RDS results in an increase in macrophages and an influx of granulocytes into airspaces [3]. These immune responses may accelerate the damage of surfactant by increased endothelial permeability and contribute to the formation of hyaline membranes and the development of chronic lung disease [1,3].

The response of premature infants with primary surfactant deficiency to intratracheally administered exogenous surfactant preparations varies widely. Proposed reasons for a poor response include deficiencies and genetic variants of surfactant protein-coding genes [4], extreme lung immaturity, delayed surfactant administration [5], mechanical ventilation [6], variations of surfactant proteins and surface-active lipids in various surfactant preparations [7], and surfactant inactivation due to increased concentrations of cytokines and other inflammatory components [1].

Surfactant influences pulmonary immunologic reactions: The surfactant proteins SP-A and SP-D regulate host immune defence and modulate inflammatory responses [8-10]. The complex of lipids and proteins in pulmonary surfactant enhances pathogen clearance and regulates adaptive and innate immune-cell functions [11]. Surfactant may decrease immunologic functions as leukocyte activation [12]. On the other hand, intratracheally administered surfactants may also induce the release of tumor necrosis factor, IL-6 and IL-8 in animal models [13,14].

DMBT1 (*Deleted *in *Malignant Brain Tumors *1), also known as glycoprotein-340 (gp-340) or salivary agglutinin (SAG), is a secreted scavenger receptor cysteine-rich (SRCR) protein mainly expressed by mucosal epithelia and glands, in particular within the human respiratory tract [15]. In the adult respiratory tract, alveolar type II cells, epithelial cells and associated glands were found to express DMBT1 [16,17]. While the steady-state DMBT1 levels appear to be low in the adult lung, immunohistochemical studies demonstrated an up-regulation upon respiratory inflammation [17]. The percentage of DMBT1-positive type II pneumocytes increases with increasing severity of inflammation.

DMBT1 secreted into the oral cavity and respiratory tract is thought to play a role in innate immune defense. The protein binds and aggregates a broad range of pathogenic bacteria as well as viruses [18-21], stimulates chemokinesis of alveolar macrophages and interacts with other components of the innate immune system (e.g. surfactant protein-D and -A) as well as secretory IgA, which plays a crucial role in adaptive mucosal immunity [16,22-25].

We hypothesized that DMBT1 could potentially be linked to the differential response to surfactant supplementation. Therefore, we studied post-mortem lung sections of neonates with respiratory distress syndrome, the effects of DMBT1 on surfactant preparations used in supplementation therapies as well as the DMBT1-level in tracheal aspirates of ventilated preterm and term infants.

## Methods

### Immunohistochemistry

We studied the expression of DMBT1 in lungs of seven newborn infants (preterm infants n = 5; full-term infants n = 2; table 1), born in 2001 – 2004, with post-mortem examination at the Institute of Pathology, University of Heidelberg, Germany.

**Table 1:** Data of patients with post-mortem examination

**Preterm infants**	**GA at birth**	**GA at death**	**Prenatal corticosteroids**	**Postnatal corticosteroids**	**Surfactant**	**RDS/IRDS**	**Acute shock lung (ASL)**
1	23	23	no	yes	yes	RDS	ASL
2	27	28	yes	yes	yes	RDS	no
3	28	30	yes	no	no	no	ASL
4	28	28	yes	no	yes	RDS	no
5	35	35	no	yes	no	IRDS	no
**Term infants**							

6	38	44	no	no	no	no	ASL
7	40	46	no	no	no	no	ASL

The analyses were approved by the responsible ethics committee. All infants showed intrapulmonary hyaline membranes because of respiratory distress syndrome in prematurity (n = 3), idiopathic respiratory distress syndrome (n = 1) and shock (n = 4; table 1). The total situation of the infants without surfactant application was so dramatic, that a surfactant application would not have changed the hopeless situation. Elevated infection parameters were diagnosed in four infants with acute bronchopneumonia, necrotizing enterocolitis with *Klebsiella pneumonia *sepsis, urosepsis and multiple organ failure after operation of cardiac vitium.

An automated Ventana Discovery stainer (Ventana Medical Systems, Tucson, USA) and a RedMap staining kit were used for immunohistochemistry with the polyclonal rabbit antiserum anti-DMBT1p84 raised against human recombinant DMBT1 (hrDMBT1). The sections were incubated with anti-DMBT1p84 antibody, diluted 1:100 in Discovery Ab Diluent, for 40 minutes followed by incubation for 30 minutes with mouse anti-rabbit IgG-AP (Santa Cruz Biotechnology, final dilution of 1:500). Immunohistochemistry with the monoclonal antibody anti-DMBT1h12 was carried out under the conditions described earlier [26]. As negative control sections were stained with equal amounts of either normal rabbit serum or anti-DMBT1p84 preincubated with hrDMBT1, or with normal mouse IgG (Santa Cruz Biotechnology). Preincubation of the polyclonal antiserum was performed by a 1:100 dilution of the anti-DMBT1p84 with a 100 μg/ml hrDMBT1 solution, followed by an incubation step at 37°C for 30 minutes prior use for the staining procedure. The control sections were negative for DMBT1 staining (data not shown).

### mRNA in situ hybridization

For *in situ *hybridization we used also formalin-fixed, paraffin-embedded tissue sections (3–4 μm). First, the sections were deparaffinized and rehydrated by xylene and a series of graded ethanols. Incubation with paraformaldehyde (PFA) for 10 minutes at 4°C was followed by blocking the endogenous peroxidase with 1% H_2_O_2 _in PBS for 20 minutes. Then the tissue sections were digested with 10 μg/ml proteinase K for 30 minutes at 37°C. Next, the sections were treated with 0.1 M triethanolamine (pH 8.0) for 20 minutes and further dehydrated by a series of graded ethanols. After prehybridization we incubated the slides with single stranded probe against the coding sequence for SRCR 6 of DMBT1 (antisense) at 47°C over night. The sense probe as negative control was used in parallel with the antisense probe on consecutive tissue sections within each experiment. The slides were treated with 2 × SSC at 47°C for 30 minutes and incubated with 50% formamide, 0.5 × SSC for 1 hour, with 50% formamide, 0.1 × SSC for 30 minutes at 47°C and finally with 0.2 × SSC for 10 minutes at room temperature.

The subsequent steps with TSA-amplification are part of the TSA™ Biotin System Kit (NEN™ Life Science Products). Blocking with TNB buffer was done for 30 minutes at room temperature. Incubation with the antibody anti-DIG-HRP, concentrated 1:100 in TNB buffer, took place for 30 minutes in a humidified chamber at room temperature. Peroxidase activity was detected by 3-amino-9-ethylcarbazole as a substrate. Finally sections were counterstained with Mayer's Hematoxylin and mounted in crystal mount (Biomeda) for microscopic examination.

### Production and purification of human recombinant DMBT1 (hrDMBT1)

The production and purification of hrDMBT1 was essentially performed as described elsewhere [27]. Briefly, the hrDMBT1 producer cell line T3 was induced with 10 μg/ml tetracycline for 48 h. Afterwards, the hrDMBT1-containing supernatant was collected and the protein was isolated by mixing the supernatant with a suspension of *S. gordonii*. Bound hrDMBT1 was eluted by using PBS buffer containing 10 mM EDTA. The eluted protein was concentrated and applied to a size-exclusion chromatography column. The purity of hrDMBT1-preparations was confirmed by SDS-PAGE and silver staining. The concentration of purified hrDMBT1 was determined with the Micro BCA Protein Assay Reagent Kit (Pierce, Rockford, USA).

### Analysis of the effect of DMBT1 on surface tension of surfactant preparations

We used two different exogenous surfactant preparations for the studies: Curosurf^® ^(extract of minced porcine lung; Nycomed Pharma GmbH, Unterschleißheim, Germany) and Alveofact^® ^(surfactant extracted from bovine lung lavage; Boehringer Ingelheim Pharma GmbH, Ingelheim, Germany). Both surfactants are lipid extracts and contain about 1% proteins (surfactant protein-B and surfactant protein-C).

The surface tension of the surfactant preparations was measured by a capillary surfactometer (Calmia Medical, Toronto, ON, Canada, [28]) as described previously [29]. Briefly, glass capillaries (0.255 mm internal diameter at its narrowest part) were filled with 0.5 μl of a surfactant suspension and subsequently we assessed the capability to keep the capillary open over a time period of 120 seconds. Distilled water (0%  of the time open) and a purified bovine surfactant (96.7% of the time open)  at 37°C served as controls [29]. The device simulates the morphology and functions of terminal conducting airways [28,29]. The "% of the time open" is used as an index of surface activity which is inversely related to the surface tension of surfactant.

Different concentrations (final concentrations: 0 – 330 μg/ml) of purified human DMBT1 recombinantly expressed in mammalian cells were added to the surfactant preparations and surface activity was measured in the presence or absence of calcium chloride (0 – 5 mM). Surface activity of each sample was measured at least three times. To examine the dependence of the inhibitory activity of hrDMBT1 on its tertiary structure we inactivated hrDMBT1 (final concentration: 200 μg/ml) by heating at 95°C for 30 minutes. Because hrDMBT1 was dissolved in PBS, we used surfactant preparations with equivalent volumes of PBS added as controls. To analyze the effect of hrDMBT1 concentration on surface tension in different surfactant preparations and the effects of calcium we used 2-way- and 1-way-ANOVAs. P values smaller than 0.05 were regarded as statistically significant.

### Tracheal aspirates

DMBT1 concentration was determined in a total of 121 tracheal aspirates of 57 ventilated preterm and term infants treated at the Perinatal Center of the University of Heidelberg in 2001 – 2004. The study was approved by the Ethics Committee. 52 of the 57 infants were preterm (mean birth weight: 1262 g, range 370 – 3915 g, mean gestational age at birth: 28 weeks, range 23 – 36 weeks). 43 of the 52 preterm infants (83%) showed RDS (mild RDS: n = 19, severe RDS: n = 24). Only 1 of the 5 term infants (mean birth weight: 3298 g, range 2420 – 3860 g, mean gestational age at birth: 39 weeks, range 37 – 40 weeks) had hyaline membranes. The tracheal aspirates were collected by instillation of sterile saline 0.9% through the endotracheal tube (<1000 g body weight (gbw) 0.5 ml; >1000 gbw 1.0 ml) and suctioned once or twice after three breaths of manual or mechanical ventilation. Subsequently, samples were additionally diluted to reach a total dilution of approximately 1:10 of the epithelial lining fluid (ELF). Samples were stored at -20°C until used for ELISA measurements.

### Determination of the DMBT1 concentration in tracheal aspirates of neonates and in surfactant preparations by ELISA (enzyme-linked immunosorbent assay)

Microtiter plates (Microlon, F-Shape, Greiner Bio-One, Frickenhausen, Germany) were coated with different dilutions of tracheal aspirates and with the surfactant preparations (Curosurf^® ^and Alveofact^®^) in coating buffer (carbonate buffer, pH 9.6) and incubated overnight at 4°C. Subsequently, the plates were washed with TBS-T and incubated with the DMBT1-specific monoclonal mouse antibody Hyb213-06 (Antibodyshop, Gentofte, Denmark, [16]) diluted 1:5000 for 1 h at room temperature. After three washes with TBS-T, the wells were incubated with a horseradish peroxidase-conjugated anti-mouse IgG (1:5000) for 1 h at room temperature. After washing, the bound enzyme was detected by adding TMB-substrate solution (125 μg/ml 3,3',5,5'-Tetramethyl-benzidine; 125 μg/ml in 0.1 M citrate buffer pH 4.5 with 0.05% (v/v) H_2_O_2_). The reaction was stopped by addition of 2 M HCl after incubation for 20 minutes in the dark. The intensity of the dye reaction was measured at 450 nm in an ELISA reader (EL800; Bio-Tec Instruments Inc.). Human recombinant DMBT1 was used as standard.

## Results

### DMBT1 expression in respiratory epithelia, glands and hyaline membranes of preterm and term infants

**Figure 1:**
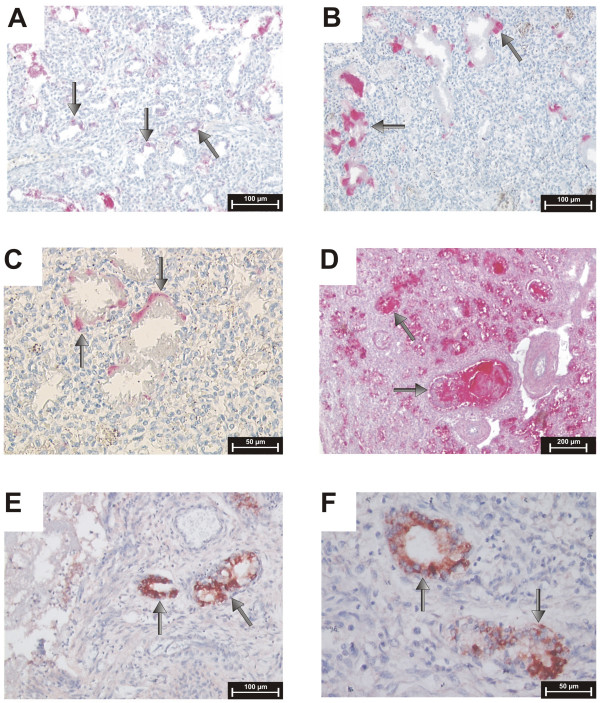
**DMBT1 expression in hyaline membranes**. Analysis of DMBT1 expression in post-mortem lung sections of preterm infants. Binding of anti-DMBT1p84 displayed as red staining (arrows). (A) Lung section of a preterm infant born at 23 weeks of gestation without infection, who died at the same day because of primary surfactant deficiency and shock after resuscitation, illustrating respiratory DMBT1 expression in hyaline membranes. (B) Lung section of a neonate with primary surfactant deficiency born at 28 weeks and dying on day 2 without infection, which demonstrates DMBT1 expression in hyaline membranes. (C) Higher lung magnification of (B). (D) Lung tissue of a preterm infant with pneumonia born at 27 weeks who died at a gestational age of 28 weeks. (E, F) mRNA *in situ *hybridization of lung section (D) confirmed the results obtained from immunohistochemical analyses.

**Figure 2:**
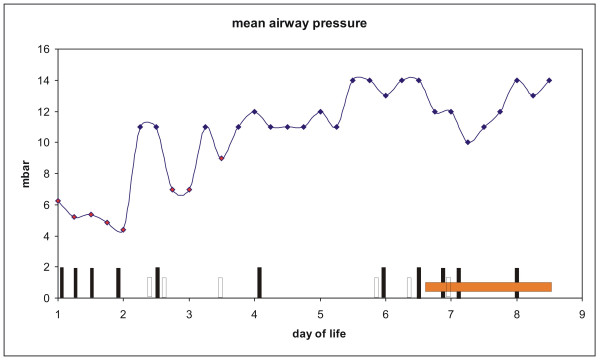
**Ventilation therapy in an infant with hyaline membranes**. Time courses of mechanical ventilation therapy and its complications in a preterm infant with bronchopneumonia that was born with 27 weeks and died at day eight (panel D-F of figure 1). The course of mean airway pressure is displayed in mbar. Red squares are mean airway pressure values under conventional ventilation (triangular airway pressure waveform) and black squares are mean airway pressure values under high-frequency ventilation (9 Hz). Black boxes: application of bovine surfactant (Alveofact^®^); empty boxes: diagnosis of pneumothorax; orange box: administration of nitrite oxygen.

The expression of DMBT1 was analyzed in postmortem lung sections of preterm and term infants by immunohistochemistry. DMBT1 expression was found in alveolar epithelial and glandular cells as well as in hyaline membranes of preterm infants with respiratory distress syndrome (RDS) and acute shock lung, and in hyaline membranes of term infants with acute shock lungs. Parallel treatments of consecutive lung sections with a DMBT1-specific monoclonal antibody (anti-DMBT1h12) and a DMBT1-specific polyclonal antiserum (anti-DMBT1p84) revealed similar staining patterns of respiratory epithelia cells and glands. The monoclonal antibody anti-DMBT1h12 gave stronger staining signals in epithelial cells, whereas the usage of anti-DMBT1p84 resulted in stronger staining signals of respiratory glands and mucus. *In situ *hybridizations confirmed the observed expression patterns. DMBT1 mainly localize on the cell surface, i.e. below/above the surfactant layer. Figure 1, panels A, B and C, show lung sections of two preterm infants (no. 1; no. 4 of table 1) who died from severe hyaline membrane disease without infection. DMBT1 expression was found in the hyaline membranes illustrated in the lung section of a preterm infant with primary surfactant deficiency and shock (Fig. 1A) as well as in the lung sections of a preterm infant with RDS caused by primary surfactant deficiency (Fig. 1B, 1C). Figure 1D presents a lung section of a preterm infant born at 27 weeks of gestation (no. 2 of table 1). The infant developed severe bronchopneumonia, surfactant deficiency, and formation of hyaline membranes. The infant received eleven doses of natural bovine surfactant preparation (Alveofact^®^), but died after 8 days due to bronchopneumonia and pulmonary interstitial emphysema with concomitant respiratory failure. The hyaline membranes stained strongly for DMBT1, which was confirmed by mRNA *in situ *hybridization revealing corresponding signals in the underlying cells (Fig. 1D – F). In Figure 2 the time course of mechanical ventilation of this preterm infant (no. 2 of table 1) with high DMBT1 levels is presented. High mean airway pressures and different forms of mechanical ventilation (synchronized intermittent mechanical ventilation, high-frequency ventilation) had to be used to reach sufficient oxygenation (Fig. 2). This was accompanied by multiple pneumothoraces and by interstitial emphysema. Multiple applications of surfactant and inhalation of nitric oxide were not able to change this fatal situation. At day 8 the preterm infant died as a consequence of insufficient oxygenation.

### DMBT1 differentially increases surfactant surface tension in vitro

**Figure 3:**
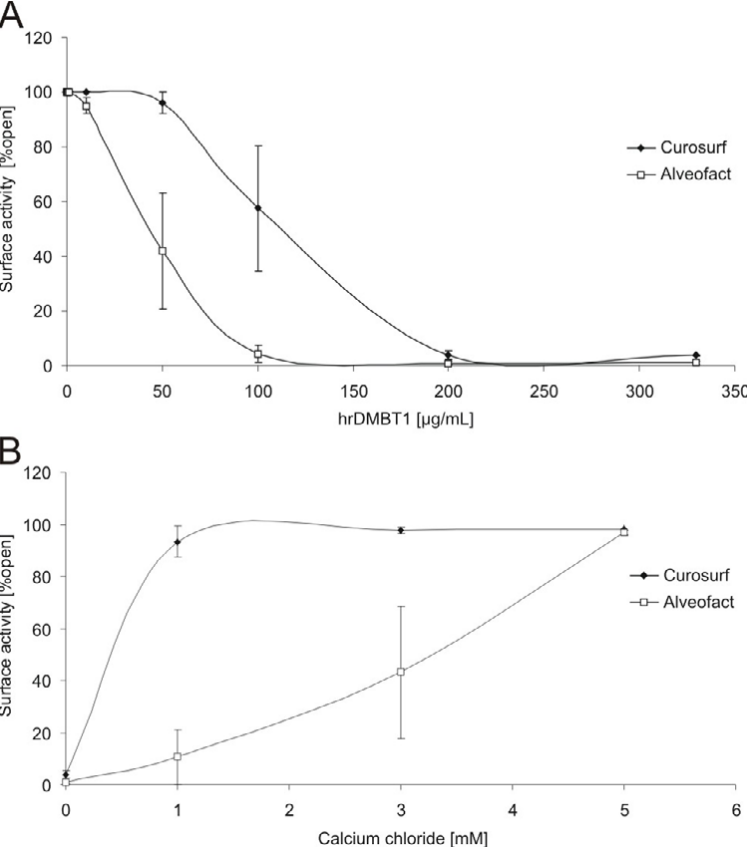
**Effects of increasing hrDMBT1 (A) and calcium (B) concentrations on surface activity of surfactant preparations**. (A) Surface activity (assessed as openness of a capillary) decreased with increasing human recombinant DMBT1 concentrations (final concentrations: 0 – 330 μg/ml) in both surfactant preparations (final phospholipid concentration 1 mg/ml). 2-way-ANOVA showed a significant influence on surface tension for surfactant preparation (P = 0.0243) and for hrDMBT1 concentration (P < 0.0001) as well as an interaction between surfactant preparation and hrDMBT1 concentration (P = 0.0363). (B) Calcium chloride antagonized the decrease of surface activity in the surfactant preparations (final phospholipid concentration 1 mg/ml) induced by hrDMBT1 (final concentration: 200 μg/ml). Analysis of variance analysis stated an influence of surfactant preparation (P = 0.0004), of hrDMBT concentration (P < 0.0001) and an interaction between surfactant preparation and hrDMBT1 concentration (P = 0.0037). Note that the surface activity of Alveofact^® ^decreased at a lower hrDMBT1 concentration than that of Curosurf^® ^(A) and that calcium antagonized the hrDMBT1 effect at 1 mM, whereas Alveofact^® ^required a calcium concentration >3 mM (B). Data are expressed as mean ± SEM.

We next aimed at exploring the effect of DMBT1 on two different surfactant preparations which are used in supplementation therapies for preterm infants. Upon ELISA measurements, there were neither detectable levels of DMBT1-homologues in bovine Alveofact^® ^nor porcine Curosurf^®^. Surfactometer measurements demonstrated that the addition of hrDMBT1 to the surfactant preparations decreased the surface activity (i.e. increased the surface tension) of both Curosurf^® ^and Alveofact^® ^in a dose-dependent manner (Fig. 3). However, the hrDMBT1 concentration needed to reduce the surface activity by 50% was about 2.5 fold higher for Curosurf^® ^(110 μg/ml hrDMBT1) than for Alveofact^® ^(45 μg/ml hrDMBT1) indicating that Alveofact^® ^is less resistant to the presence of DMBT1 (Fig. 3A). Analysis by 2-way-ANOVA showed a significant influence on surface tension depending on the surfactant preparation (P = 0.0243) and on the hrDMBT1 concentration (P < 0.0001) as well as an interaction between surfactant preparation and hrDMBT1 concentration (P = 0.0363), which confirmed that the concentration influence is different in the two surfactant preparations. Therefore, each surfactant preparation was investigated by a 1-way-ANOVA. For Alveofact^® ^and Curosurf^®^, a highly significant influence of hrDMBT1 on the surface tension could be shown (P < 0.0001 or P = 0.0001, respectively).

The decrease of surface activity induced by 200 μg/ml hrDMBT1 was completely abolished by 1 mM CaCl_2 _for Curosurf^®^, whereas 5 mM CaCl_2 _was necessary to obtain a comparable effect for Alveofact^® ^(Fig. 3B). Also in this setting the 2-way-ANOVA pointed to an influence of surfactant preparation (P = 0.0004), of hrDMBT1 concentration (P < 0.0001) and an interaction relation between surfactant preparation and hrDMBT1 concentration (P = 0.0037). One-way-ANOVA revealed a significant influence of hrDMBT1 concentration in Alveofact^® ^as well as in Curosurf^® ^(P = 0.0034 and P < 0.0001, respectively). These data demonstrate that hrDMBT1 has a differential effect on the surface tension of different surfactants *in vitro*, which can be antagonized by the addition of calcium chloride.

**Figure 4:**
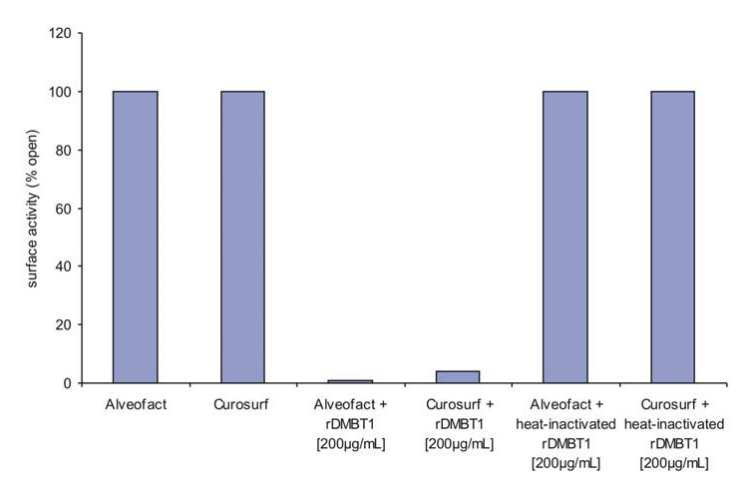
***In vitro *surfactometer measurements of surface activity**. Pure surfactants (final phospholipid concentration 1 mg/ml) showed high surface activity (100% open). Human recombinant DMBT1 (final concentration: 200 μg/ml) decreased the surface activity of Alveofact^® ^and Curosurf^® ^(final phospholipid concentration 1 mg/ml). Heat-inactivated hrDMBT1 had no effect on surface activity.

To assess the dependence of the inhibitory activity of DMBT1 on its tertiary structure, heat-inactivated hrDMBT1 (final concentration: 200 μg/ml) was added to Curosurf^® ^or Alveofact^®^, both with a final phospholipid concentration of 1 mg/ml. No inhibitory effect of heat-treated hrDMBT1 was found on the surface activity of the used porcine and bovine surfactant preparations (Fig. 4).

### DMBT1 levels in tracheal aspirates of preterm and term infants

**Figure 5:**
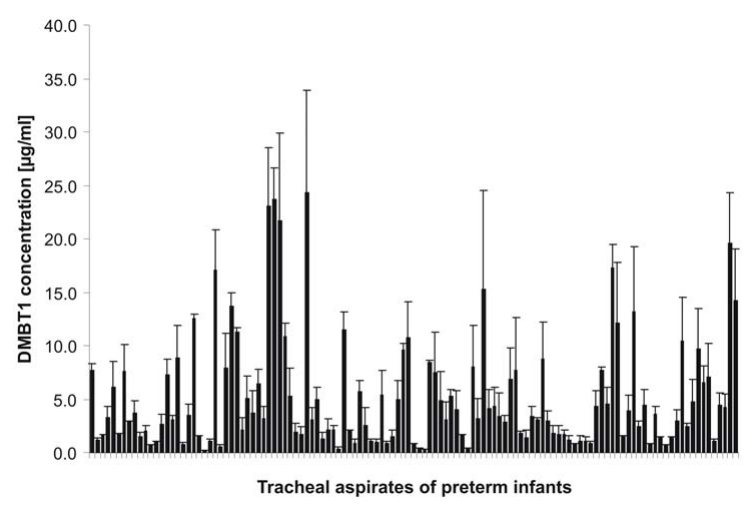
**DMBT1 concentration in 1:10 diluted tracheal aspirates of preterm and term infants without correction for epithelial lining fluid dilution**. The DMBT1 concentration in 121 tracheal aspirates of 57 ventilated preterm and term infants was analyzed by ELISA using the monoclonal antibody Hyb213-06. The mean DMBT1 concentration was 5.2 ± 1.8 μg/ml. Due to our sampling procedure of tracheal aspirates and due to an additional dilution after sampling, we estimate the volume of epithelial lining fluid in our tracheal aspirates to be at least one-tenth of the recovered volume.

Surfactometer measurements indicate that DMBT1 is able to increase the surface tension of two different surfactant preparations, which would have a negative impact on the treatment of preterm infants. In order so see whether the DMBT1 concentrations used in the *in vitro *experiments match to the concentrations present *in vivo*, we analyzed 121 tracheal aspirates (TAs) of 57 preterm and term infants in ELISA (Fig. 5). The 1:10 diluted TAs contained a mean DMBT1 concentration of 5.2 ± 1.8 μg/ml with a lowest DMBT1 concentration of 0.2 μg/ml and a highest of 25 μg/ml, respectively (Fig. 5). In summary 47% of the analyzed tracheal aspirates showed a DMBT1 concentration of more than 15 μg/ml.

## Discussion

Insufficient surfactant production or inactivation of surfactant is a frequent cause for mechanical ventilation and surfactant application in neonatology. Surfactant deficiency results in alveolar collapse and impaired gas exchange associated with the requirement of high pressure in mechanical ventilation and with the appearance of pneumothoraces. We studied the expression of the secreted scavenger receptor cysteine-rich protein DMBT1 in ventilated preterm and term infants. Our results demonstrate that respiratory epithelial cells and glands of preterm and term neonates expressed DMBT1. We found out that DMBT1 was located in hyaline membranes of preterm infants with RDS and acute shock and of term infants with acute shock lungs. DMBT1 was mainly found on the surface, i.e. below/above the surfactant layer. DMBT1 is thought to be a critical factor of innate defence mechanisms, therefore its strong expression in preterm infants is in agreement with the strong immune response induced by RDS [1,2].

In neonatology the introduction of surfactant application was a great success in the treatment of neonates and contributes to survival of very low birth weight infants. Our investigations revealed that DMBT1 (hrDMBT1) is able to inactivate two commercially available surfactant preparations (Curosurf^®^; Alveofact^®^) in a dose dependent manner (Fig. 3). A 50% reduction of surface activity was observed in Alveofact^® ^by 45 μg/ml hrDMBT1 and in Curosurf^® ^by 110 μg/ml hrDMBT1 indicating that Alveofact^® ^is less resistant to the presence of DMBT1. The presence of DMBT1 in epithelial lining fluid of ventilated preterm and term infants could be confirmed by ELISA measurements of tracheal aspirates (Fig. 5). DMBT1 concentrations determined in 1:10 diluted tracheal aspirates by ELISA were in the range of 0.2 – 25 μg/ml, with a mean concentration of 5 μg/ml DMBT1. A mean DMBT1-concentration of 130 ± 30 ng/ml has been reported in unconcentrated bronchoalveolar lavage (BAL) of healthy adults [21]. However, a yield of 0.5 μg DMBT1^gp340 ^per ml BAL from adult patients with alveolar proteinosis has been described and indicates much higher DMBT1-levels in the diseased lung of adults [24]. Both the DMBT1 concentrations measured in BAL fluid [21] and in preterm tracheal aspirates do not reflect the "real" concentration in the epithelial lining fluid (ELF) because of dilutions during the lavage and aspirate procedure. The volume of epithelial lining fluid recovered by lavage in adults has been estimated to be one-hundreth of the recovered lavage volume [30]. Due to our sampling procedure of tracheal aspirates and due to an additional dilution after sampling, we estimate the volume of epithelial lining fluid in our tracheal aspirates to be at least one-tenth of the recovered volume. This would mean that the local DMBT1 level in epithelial lining fluid of ventilated preterm infants could indeed reach a level in which an inactivation of applied surfactant preparations might occur. Therefore, DMBT1 levels in preterm infants might be of importance for the clinical effectiveness of surfactant preparations.

At the present state of the art, it is unclear by which mechanisms DMBT1 alters surfactant function. It is conceivable that DMBT1 may inhibit surfactant absorption to the surface of terminal conducting airways and alveoli. This mechanism has been proposed for surfactant inactivation by serum proteins such as albumin and fibrinogen [31]. Addition of albumin or fibrinogen to lung surfactant changes the characteristic surfactant morphology from a lamellar rod-like structure with open ends into spherical structures with loss of their open ends [32].

However, the addition of calcium to the preparations could partly revert the negative effect of hrDMBT1 on the surface activity (Fig. 3B). Whereas the addition of 1 mM CaCl_2 _overcame the increase of surface tension by 200 μg/ml hrDMBT1 in Curosurf^®^, it required more than 3 mM CaCl_2 _to obtain a comparable effect for bovine Alveofact^®^. Bernhard et al. showed improved function of Alveofact^® ^and Curosurf^® ^when increasing calcium to nonphysiologic concentrations of 3 mM and 4 mM, respectively [33]. Calcium has previously been shown to improve spreading and adsorption of surfactant [34]. This effect of calcium can probably be explained by increased aggregation of surfactant lipids, thereby forming larger and more effective aggregates [33,35-37]. A beneficial effect of calcium on the surface tension of Curosurf^® ^has also been found in incubation experiments with meconium [34]. Surfactant inactivation by meconium was also associated with altered surfactant morphology [38,39] which could be improved by increased calcium concentrations [34].

Several differences between the two surfactants may contribute to the increased resistance of Curosurf^® ^to hrDMBT1 when compared with Alveofact^®^. Curosurf^® ^contains higher amounts of phosphatidylinositol, phosphatidylethanolamine, sphingomyelin and phosphatidylserine, and higher concentrations of polyunsaturated fatty acid-containing phospholipids and of plasmalogens than Alveofact^® ^[7]. *In vitro *Curosurf^® ^is a stronger inhibitor of induced neutrophil activation than Alveofact^® ^[40]. Meconium induced a significant increase in minimum surface tension at a concentration of ≥0.02 mg/ml for Alveofact^® ^and at ≥0.04 mg/ml for Curosurf^® ^[41].

Decreased surface activity of surfactant indicates impaired ability of surfactant to keep the terminal conducting airways and alveoli open. Up-regulated concentrations of DMBT1 may contribute to oxygenation difficulties by either interfering with the function of the endogenous surfactant and/or leading to a decreased efficacy of exogenously added surfactant preparations, such as Curosurf^® ^and Alveofact^®^. This raises the possibility that DMBT1, in addition to its role in innate host defense, may function by influencing the surface tension of pulmonary surfactant.

## Conclusion

Our results demonstrate that preterm and full-term infants express DMBT1 in the lung. Respiratory DMBT1 may modulate surface activity of exogenous surfactant in an unfavorable manner, thereby possibly contributing to oxygenation defects. Thus, inhibitors of these DMBT1-mediated effects could represent useful agents to counteract neonatal lung disease, intractable surfactant deficiency and respiratory failure in addition to drugs already used in neonatal intensive care units such as prenatal corticosteroids and surfactant. To confirm such relationships follow-up studies are required that address potential correlations between increased DMBT1 levels in bronchoalveolar fluid and inactivation of exogenously added surfactant preparations *in vivo*.

## Competing interests

The author(s) declare that they have no competing interests.

## Authors' contributions

HM designed research, performed research, analyzed data, and wrote the paper; CE performed production and purification of human recombinant DMBT1 and was involved in the experimental design and performance; MR participated in immunohistochemical analysis and mRNA *in situ *hybridization of human tissue; BMH contributed clinical samples and participated in analyzing data of immunohistochemistry and mRNA *in situ *hybridization, NG participated in analyzing immunohistochemical data; CW performed statistical analysis, DH participated in analysis of surfactant function and assisted in writing the paper; MG participated in analysis of surfactant function; MH assisted in performing research; AP assisted in performing research; JM assisted in designing research, performing research, analyzing data; JP assisted in performing research. The authors read and approved the manuscript.
